# PELI1 and EGFR cooperate to promote breast cancer metastasis

**DOI:** 10.1038/s41389-023-00457-3

**Published:** 2023-02-25

**Authors:** Jie Qi, Guangsen Xu, Xiaoxia Wu, Chunhua Lu, Yuemao Shen, Baobing Zhao

**Affiliations:** 1 Key Lab of Chemical Biology (MOE), School of Pharmaceutical Sciences, Cheeloo College of Medicine, Shandong University, 250012 Jinan, Shandong China; 2 NMPA Key Laboratory for Technology Research and Evaluation of Drug Products, School of Pharmaceutical Sciences, Cheeloo College of Medicine, Shandong University, 250012 Jinan, Shandong China; 3 Department of Pharmacology, School of Pharmaceutical Sciences, Cheeloo College of Medicine, Shandong University, 250012 Jinan, Shandong China

**Keywords:** Breast cancer, Metastasis, Post-translational modifications, Cancer therapy

## Abstract

Pellino-1 (PELI1) is an E3 ubiquitin ligase acting as a key regulator for the inflammation and autoimmunity via the ubiquitination of the substrate proteins. There is increasing evidence to support that PELI1 functions as an oncoprotein in tumorigenesis and metastasis. However, the molecular mechanism underlying the high expression and oncogenic roles of PELI1 in cancers remains limited. Herein, we revealed a novel regulation mechanism by which PELI1 and EGFR cooperate to promote breast cancer metastasis. EGFR is positively correlated with PELI1 expression in breast cancers, and its activation led to the phosphorylation of PELI1 at Tyr154 and Thr264, which subsequently activated its E3 ubiquitin ligase. Simultaneously, PELI1 physically interacted with and enhanced the stability of EGFR via the K63-linked polyubiquitination in reverse. The co-inhibition of the PELI1-EGFR showed synergetic effect to repress breast cancer metastasis. Furthermore, we identified a compound S62 as a small molecule disruptor of PELI1/EGFR that effectively repressed breast cancer metastasis. Our study not only uncovered the emerging roles of PELI1/EGFR interaction in the progression of breast cancer, but also provided an effective strategy for the inhibition of metastasis in breast cancer.

## Introduction

Breast cancer is the second frequent malignancy in women, in which triple negative breast cancer (TNBC) especially has a high metastasis rate [1, 2]. Metastasis as a representative hallmark of most cancers is primarily responsible for the death from breast cancer [3, 4]. It is a cellular process through several mechanisms including epithelial-mesenchymal transition (EMT), by which breast cancer cells cross the surrounding basement membrane and enter the vascular system to spread into distant organs, leading to resistance to therapy and treatment failure [5, 6].

Pellino-1 (PELI1) is an E3 ubiquitin ligase as well as other three members PELI2, PELI3a and PELI3b [7]. Pellino family are highly conservative in primary structure, possessing a C-terminal RING-like domain and a forkhead-associated (FHA) domain. RING-like domain confers E3 ubiquitin ligase activity, while FHA domain is responsible for its interaction with the substrate proteins. PELI1 has been demonstrated to function as a key regulator for the inflammation and autoimmunity via the ubiquitination of the substrate proteins, including Toll-like receptors, IL-1 receptor and T cell receptors [8, 9]. There is increasing evidence to support that PELI1 functions as an oncoprotein in tumorigenesis and metastasis [10–16]. PELI1 promoted lymphomagenesis by regulating B cell chronic lymphocytic leukemia (BCL6) via the K63-linked polyubiquitination [10]. It has also been demonstrated that PELI1 contributed to the EMT of lung cancer by the ubiquitination-mediated stabilization of SNAIL and SLUG [11]. Similarly, we previously found that PELI1 is highly expressed and promotes the progression of TNBC through induction of SNAIL/SLUG [12].

Epidermal growth factor receptor (EGFR), a glycoprotein that belongs to transmembrane tyrosine kinase receptor ErbB family, is involved in a various of physiological processes including cell proliferation and migration of tumors [17–19]. Upon ligand binding such as EGF, amphiregulin, and transforming growth factor α, EGFR dimerizes with itself or other ERBB members, triggering its autophosphorylation of the cytoplasmic domain that activate the intracellular signaling cascades [20, 21]. Given that the gain-of-function with activating mutations of the *EGFR* gene occurs in various malignancies, EGFR has been regarded as an important therapeutic target for these tumors [22–24]. At present, a consistent benefit in favor of EGFR-targeted therapeutic approaches is observed across studies, including EGFR antibody-based treatments as well as its tyrosine kinase inhibitors (TKI) [25–28]. However, resistance to EGFR-targeted treatments is also commonly acquired due to the emergence of novel EGFR mutations, feedback regulatory loops and altered endocytosis/recycling of EGFR [29, 30]. Thus, simultaneous targeting EGFR independent of its mutation and basic cellular processes has been proposed as a potential therapeutic strategy [31, 32].

One previous study implied that EGFR was involved in the regulation of PELI1 in lung tumorigenesis beyond the SNAIL/SLUG [11], however, the underling mechanisms are totally unclear. Here, we found that PELI1 is positively correlated with EGFR in breast cancers. PELI1 physically interacted with EGFR to cooperate to promote breast cancer metastasis. The co-inhibition of the PELI1-EGFR effectively repressed breast cancer metastasis and enhanced the sensitivity of EGFR-TKI. Our study provides mechanistic insights and therapeutic interventions for breast cancer metastasis.

## Results

### PELI1 is positively correlated with EGFR in breast cancers

**Fig. 1:**
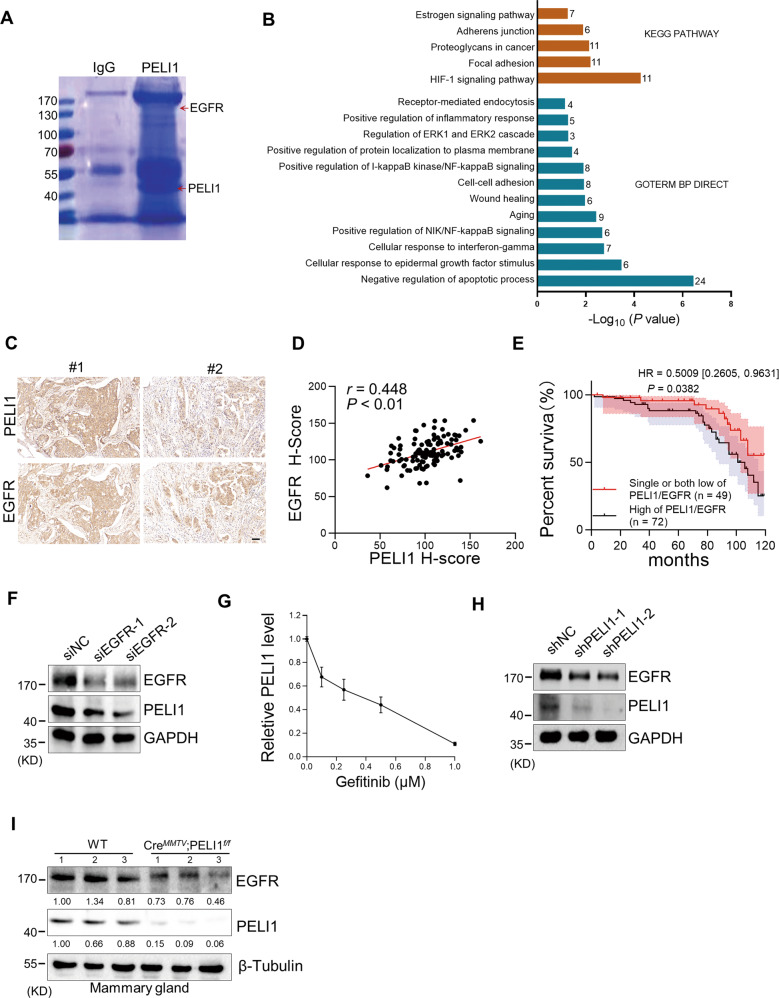
The levels of PELI1 and EGFR were positively correlated in human breast cancer. **A** MDA-MB-231 cells overexpressing PELI1 were lysed and immunopurified with normal IgG and anti-PELI1 antibody respectively. Then the complex was resolved by SDS–PAGE followed by Coomassie Blue staining. The distinct bands were analyzed by MS. Red arrows indicated the identified PELI1 and EGFR. **B** Representative biological processes and signaling pathways significantly enriched from proteins identified from Co-IP with PELI1 in MDA-MB-231 cells. GO and KEGG analysis were performed using DAVID bioinformatics database. The number of enriched proteins in relative terms was shown in each bar. **C**, **D** IHC analysis of PELI1 and EGFR in the tissues from breast cancer patients’ tissues microarray. Representative images of IHC staining (**C**) and the correlation rate (**D**) by Pearson’s test is shown (*N* = 121, scale bar, 50 μm). **E** Overall survival rates were determined by Kaplan–Meier analyses of indicated groups. Hazard ratio (HR) and *P* values (log rank *P*) are shown. **F** Western blotting analysis of PELI1 protein levels in MDA-MB-231 cells with EGFR knockdown. GAPDH was used as loading control. **G** ELISA analysis of the change of PELI1 in MDA-MB-231 cells with Gefitinib. **H** Western blotting analysis of EGFR protein levels in MDA-MB-231 cells with PELI1 knockdown. GAPDH was used as a loading control. **I** Sample immunoblotting showed the levels of PELI1 and EGFR proteins in mammary gland from indicated transgene mice. β-Tubulin was used as a loading control.

Considering our previous findings that PELI1 showed higher expression in breast cancer than adjacent tissues, we examined whether PELI1 is commonly abundant in other cancers by a multiple tumor tissue microarray assay, including colon cancer, lung cancer, rectum cancer, breast cancer and prostate cancer. Interestingly, comparable high-expression of PELI1 was observed in these cancers relative to breast cancer, suggesting that PELI1 is an epigenetically regulated pan-cancer oncogene (Fig. S1A, B). To explore the underlying mechanisms involved in the breast cancer progression mediated by PELI1, we performed co-immunoprecipitation assays for PELI1 in MDA-MB-231 cells followed by mass spectrometry (Fig. 1A). Many candidates of binding partners of PELI1 were identified, which were significantly enriched into distinct signaling besides posttranslational modification (Fig. 1B and Table S1). Notably, EGFR was also identified by its six specific peptides, and involved in most of these enriched signaling (Fig. S1C), indicating that EGFR is one of the interacting proteins of PELI1.

Given that EGFR is closely related to the tumorigenesis, we analyzed the PELI1 and EGFR expression using the GEPIA database, and found that PELI1 was positively correlated with EGFR expression in multiple cancers besides breast cancers (Fig. S1D). We also examined the PELI1 and EGFR protein levels in breast cancer tissue microarray by IHC, and found a highly positive correlation (Fig. 1C, D). Furthermore, the highest co-expression was also detected in breast cancer cells among five kinds of cancer cell lines, including MDA-MB-231, HCT-116, H1299, SKVO3 and HT-29 cells (Fig. S1E). Notably, breast cancer patients with both high expressions showed a lower survival rate than the ones harboring their single high or both low expressions (Fig. 1E).

To further confirm their correlations, we silenced *EGFR* expression with siRNA, and found that PELI1 protein levels were dramatically reduced after EGFR knockdown in MDA-MB-231 cells (Fig. 1F). Furthermore, the expression of PELI1 was also markedly suppressed by EGFR inhibitors such as Gefitinib and Lapatinib, as indicated by the immunoblotting and enzyme-linked immunosorbent assay (Figs. 1G and S1F, G). These data indicated that EGFR regulates the expression of PELI1.

We next examined whether PELI1 reversely regulates EGFR expression. PELI1 knockdown with shPELI1 lentivirus led to a remarkable decrease of EGFR in MDA-MB-231 cells (Fig. 1H). Furthermore, EGFR expression was further evaluated in *PELI1* knockout background. PELI1^flox/flox^ mice were mated with MMTV-Cre mice to generate mammary conditional knockout mice (Cre^*MMTV*^; PELI1^*f/f*^). As expected, EGFR expression were markedly decreased in mammary tissue from Cre^*MMTV*^PELI1^*f/f*^ mice compared to WT group (Fig. 1I). These results confirmed that PELI1 is positively correlated with EGFR expression.

### PELI1 physically interacted with EGFR

**Fig. 2:**
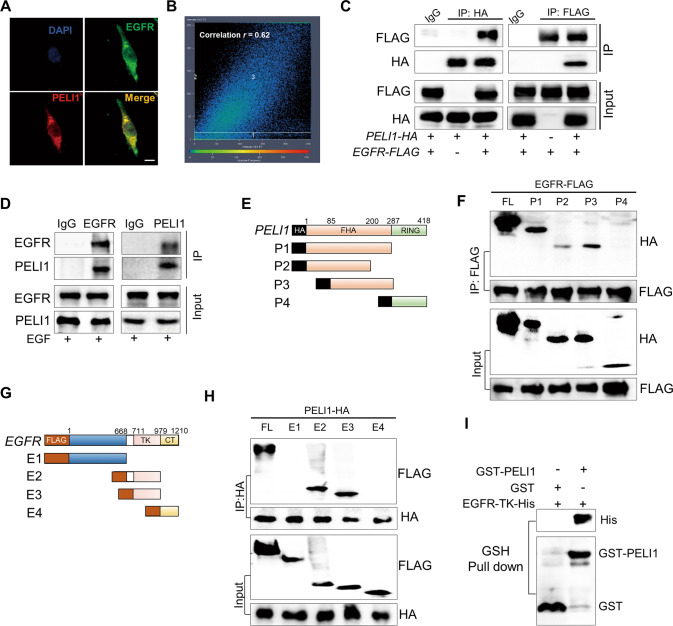
PELI1 directly interacted with EGFR. **A**, **B** IF staining showed co-localization between PELI1and EGFR in MDA-MB-231 cells (scale bar, 10 μm). The co-localization correlation rate of PELI1 and EGFR in **A** is shown (**B**). **C** The interaction between PELI1 and EGFR was detected by Co-IP assay. HEK293T/17 cells were co-transfected with pCDNA3.1-HA-*PELI1* and pLVX-FLAG-*EGFR* plasmids. The cells were harvested and subjected to immunoprecipitated with anti-HA and anti-FLAG antibodies respectively. Similar Co-IP analysis with normal IgG were performed as control. **D** Co-IP analysis of endogenous PELI1 and EGFR in MDA-MB-231 cells. The cell extracts were immunoprecipitated with anti-IgG, anti-EGFR and anti-PELI1 antibodies, respectively, upon EGF (100 ng/ml) stimulation. **E** The sketch map of the deletion mutant regions of PELI1. **F** Co-IP analysis of PELI1 mutants binding to EGFR. HEK293T/17 cells were co-transfected with *PELI1* deletion mutants (HA tagged) and pLVX-FLAG-*EGFR* plasmids, and IP analysis was performed with anti-FLAG antibody. **G** The sketch map of the deletion mutant regions of EGFR. **H** Co-IP analysis of EGFR regions binding to PELI1. HEK293T/17 cells were co-transfected with *EGFR* deletion mutants (FLAG-tagged) and pCDNA3.1-HA-*PELI1* plasmids, and IP analysis was performed with ani-HA antibody. **I** GST-pull down assay of the direct correlation between PELI1 and EGFR-TK.

Consistent with the findings from IP-MS, we also found that PELI1 was closely colocalized with EGFR by immunofluorescence staining in MDA-MB-231 cells (Fig. 2A, B). To further confirm the interaction between PELI1 and EGFR, we transfected pcDNA3.1-HA-*PELI1* and pLVX-FLAG-*EGFR* constructs into HEK293T/17 cells, followed by the immunoprecipitation with anti-HA and anti-FLAG antibodies respectively. The Co-IP results showed that PELI1 and EGFR were reciprocally coimmunoprecipitated with each other, indicating a physical interaction between both of them (Fig. 2C). This is further supported by the Co-IP assays of endogenous PELI1 and EGFR in MDA-MBA-231 cells (Fig. 2D).

To determine the domains that are responsible for their interactions, we constructed a series of truncated forms of EGFR and PELI1 (Fig. 2E, G). Correlatively, EGFR showed a specific interaction with FHA domain of PELI1 (Fig. 2F), while the intracellular tyrosine kinase (TK) domain of EGFR is responsible for the interaction with PELI1 (Fig. 2H). This was further confirmed by the pull-down assays (Fig. S2), which showed that TK-domain-truncated EGFR (His tag) were specifically retained in the presence of PELI1 (Fig. 2I).

### PELI1 stabilized EGFR through K63-linked ubiquitination

**Fig. 3:**
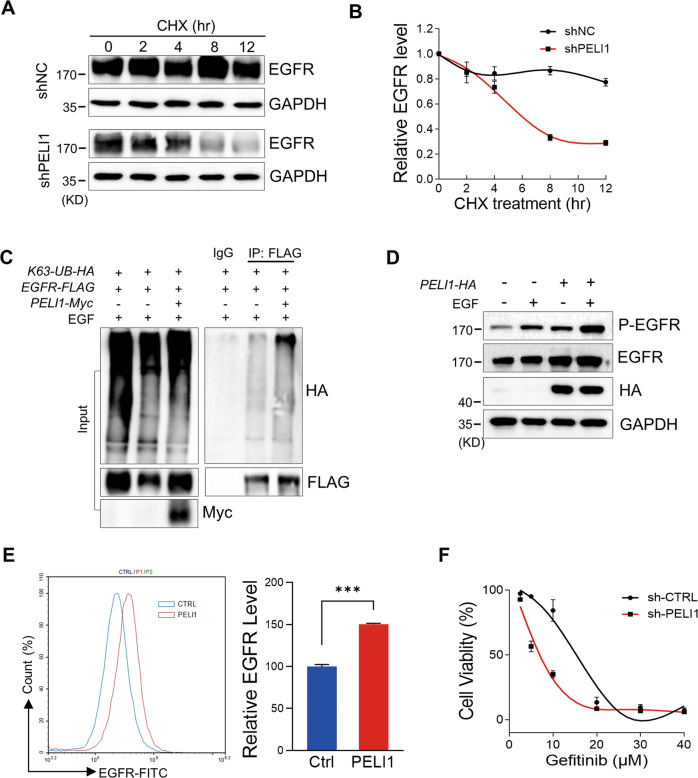
PELI1 ubiquitinated EGFR and inhibited its degradation. **A**, **B** Western blotting analysis of EGFR protein levels in MDA-MB-231 cells with PELI1 knockdown upon the treatment of CHX (10 μg/ml). EGFR levels were normalized to the change of GAPDH (*N* = 3). **C** Western blotting analysis of K63-linked polyubiquitination of EGFR immunoprecipitated from HEK293T/17 cells co-overexpressing PELI1 and EGFR upon EGF stimulation (100 ng/ml). **D** Western blotting analysis of EGFR proteins in MDA-MB-231 cells with or without PELI1 overexpression upon EGF (100 ng/ml) stimulation. **E** Flow cytometric analysis of membrane EGFR levels in MDA-MB-231 cells with PELI1 overexpression upon EGF (100 ng/ml) stimulation (*N* = 3). ***P < 0.001. **F** Effect of PELI1 knockdown on the cell viability of MDA-MB-231 cells was detected upon the treatment of Gefitinib.

Considering PELI1 as an E3 ubiquitin ligase and its interaction with EGFR, we investigated whether EGFR is a potential target for PELI1. We first performed cycloheximide (CHX) treatment in MDA-MB-231 cells transfected with shScramble and shPELI1 lentivirus, respectively. CHX, an inhibitor of protein synthesis, led to a mild decrease of EGFR in MDA-MB-231 cells. On the contrary, PELI1 knockdown accelerated the time-dependent decrease of EGFR proteins, indicating that PELI1 protects EGFR from the degradation (Fig. 3A, B).

We next defined the type of polyubiquitinated linkages attached to EGFR mediated by PELI1. K48-linked ubiquitination are the most common chain type and target proteins for proteasomal degradation, while K63-linked ubiquitination has many well-studied non-degradative roles on the substrates [33, 34]. HEK293T/17 cells were transfected with HA-tagged K63-linked ubiquitin and FLAG-tagged EGFR combined with PELI1 overexpression, followed by the immunoprecipitation for EGFR and subsequent immunoblotting analysis of its ubiquitination. Interestingly, overexpression of PELI1 promoted K63-linked ubiquitination of EGFR but it failed to detect any increase of K48-linked ubiquitination of EGFR upon enforced expression of PELI1 (Figs. 3C and S3A, B). Furthermore, we found that enforced expression of PELI1 led to the increase of EGFR in MDA-MB-231 cells (Fig. 3D). The plasma membrane EGFR was also upregulated as indicated by the flow cytometry analysis (Fig. 3E).

EGFR plays roles in malignant transformation and cancer metastasis, and its dysregulated activation has been regarded as multifaceted hallmarks of cancer cells [35, 36]. Thus, we further examined the contributions of PELI1 to aberrant EGFR signaling in cancers. As expected, Gefitinib, an inhibitor of EGFR approved for the clinical treatment of cancers [37], induced a dose-dependent reduction of the cell viability in MDA-MB-231 cells, and this is attenuated by the enforced expression of PELI1 (Fig. S3C). On the contrary, PELI1 knockdown enhanced the sensibility of EGFR inhibitor against MDA-MB-231 cells (Fig. 3F). To further confirm the gains of PELI1 inhibition in the EGFR-targeting therapy, we performed similar cell viability assays in two lung cancer cell lines NCI-H1650 and NCI-H1975 with oncogenic activations of EGFR. In line with the findings in breast cancers, *EGFR* expression is correlated with the highly expressed *PELI1* in these two lung cancer cell lines (Fig. S3D). Similarly, PELI1 knockdown also enhanced the sensibility of EGFR inhibitor against NCI-H1650 and NCI-H1975 (Fig. S3E, F). In addition, immunoblotting demonstrated that EGFR signaling and EMT related proteins (Vimentin and SNAIL) were considerably downregulated after PELI1 knockdown in these two lung cancer cell lines (Fig. S3G). Taken together, these data demonstrated that PELI1 mediated EGFR stability through K63-linked ubiquitination to be involved in the aberrant EGFR signaling in cancers.

### EGFR activation led to the phosphorylation and activation of PELI1

**Fig. 4:**
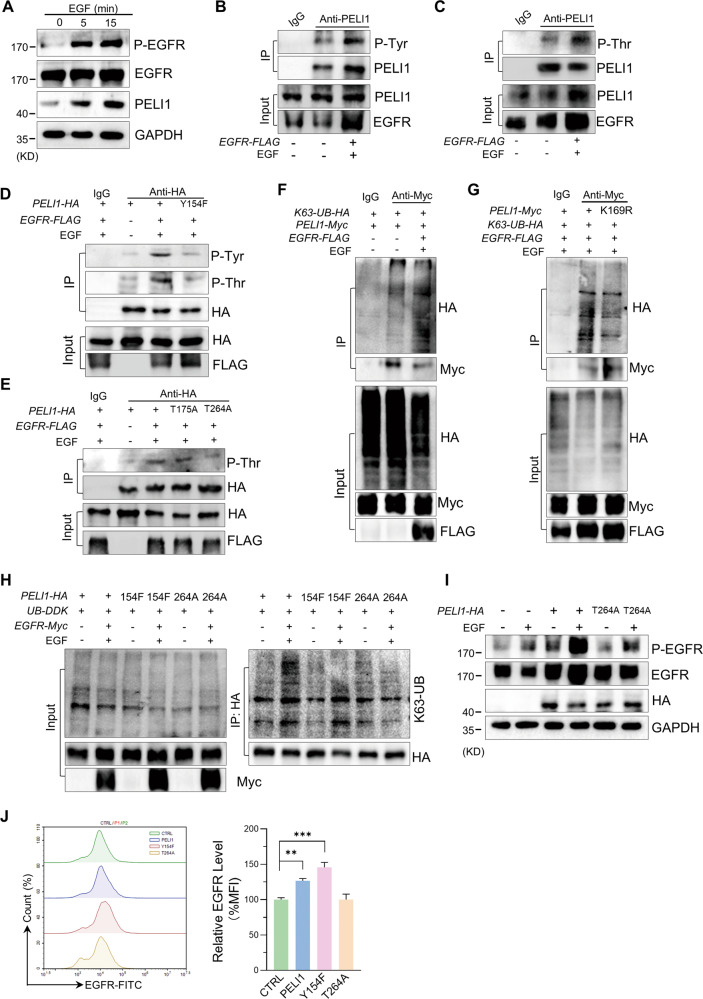
EGFR phosphorylated PELI1 leading to its K63-linked auto-ubiquitination. **A** Western blotting analysis of the levels of indicated proteins in response to EGF (100 ng/ml). **B**, **C** Western blotting analysis of the tyrosine (**B**) and threonine (**C**) phosphorylation of PELI1 with EGFR overexpression upon EGF stimulation (100 ng/ml) in MDA-MB-231 cells. **D**, **E** Western blotting analysis of the tyrosine or threonine phosphorylation of PELI1 immunoprecipitated from HEK293T/17 cells with overexpression of the indicated *PELI1* mutants. **F** Western blotting analysis of the K63-mediated polyubiquitination of PELI1 immunoprecipitated from HEK293/17 cells co-overexpressing with EGFR and PELI1 upon EGF (100 ng/ml) stimulation. **G** Similar with F excluding overexpressing PELI1 mutant (K169F). **H** PELI1 was immunoprecipitated from HEK293T/17 cells transfected with *PELI1*, *PELI1*-Y154F or *PELI1*-T264A plasmids, followed by Western blotting analysis of the K63-mediated polyubiquitination. **I** Western blotting analysis of EGFR and phospho-EGFR in MDA-MB-231 cells overexpressed *PELI1* or *PELI1*-T264A upon EGF (100 ng/ml) stimulation. **J** Flow cytometric analysis of membrane EGFR in MDA-MB-231 cells with overexpression of *PELI1*, *PELI1*-Y154F or *PELI1*-T264A (*N* = 3). **P < 0.01, ***P < 0.001. All *P* values were determined by unpaired two-tailed Student’s *t* test.

To further determine the regulatory effect of EGFR on PELI1, we evaluated the expression of PELI1 upon EGF stimulation in MBA-MB-231 cells. The immunoblotting showed that PELI1 protein levels were significantly increased with EGFR activation indicated by the upregulated phosphorylation (Fig. 4A), which coinciding with an increase of the plasma membrane EGFR (Fig. S4A). Notably, the rapid upregulation of PELI1 expression in MBA-MB-231 cells after 5 min EGF stimulation, suggested that EGFR regulated the stability of PELI1 proteins.

Given that the phosphorylation of PELI1 is required for its activation and subsequent autoubiquitylation [38], we thus analyzed the phosphorylation status of PELI1 upon EGF stimulation in MBA-MB-231 cells. Endogenous PELI1 proteins were immunoprecipitated from MBA-MB-231 cells, followed by immunoblotting for the phosphorylated tyrosine residues and threonine residues respectively (Fig. 4B, C). The elevated levels of phosphorylated Tyr/Thr in MBA-MB-231 cells with EGFR overexpression and activation indicated that EGFR is an upstream phosphokinase of PELI1. We then performed phosphoamino acid analysis and identified three novel phosphorylation sites of PELI1, including Tyr154, Thr175 and Thr264 (Fig. S4B, C).

To further define the phosphorylation sites of PELI1, we constructed three corresponding inactive mutants of PELI1, containing a single mutation as Thr175 (T175A) and Thr264 to alanine (T264A), and Tyr154 to phenylalanine (Y154F) respectively. Interestingly, Y154F mutant failed to show EGFR-induced increase of phosphorylated Tyr/Thr compared to that of WT form of PELI1 (Fig. 4D). Furthermore, T264A mutant but not T175A completely abolished the phosphorylation of Thr upon the EGFR activation (Fig. 4E). These data suggested that PELI1 was phosphorylated at Tyr154 upon EGFR activation that led to its phosphorylation at Thr264.

We then determined whether PELI1 undergoes the autoubiquitination after the phosphorylation induced by EGFR. Indeed, overexpression of EGFR promoted K63-linked ubiquitination of PELI1 (Fig. 4F). However, it failed to show any K48-linked ubiquitination of PELI1 upon enforced expression of EGFR (Fig. S4D). Additionally, we also identified the ubiquitination site of PELI1 as Lys 169 via IP-MS analysis (Fig. S4E, F). To further confirm it, we transfected a mutant form PELI1 (K169R) and EGFR into HEK293T/17 cells and immunoblotting demonstrated that this lysine residue is mainly responsible for the K63-linked ubiquitination of PELI1 (Fig. 4G).

In line with the previous study to show the roles of the phosphorylation of PELI1 in its activation [38], we found that overexpression of PELI1-Y154F, as well as PELI1-T264A, reversed the K63-linked ubiquitination of PELI1 mediated by EGFR (Fig. 4H). Indeed, overexpression of PELI1-T264A also failed to show the increase of intracellular EGFR and the plasma membrane EGFR in MDA-MB-231 cells (Fig. 4I, J). Therefore, these results revealed that EGFR activation led to the phosphorylation of PELI1 at Y154 residue that is required for the activation of PELI1.

### Inhibition of PELI1 and EGFR suppressed breast cancers metastasis

**Fig. 5:**
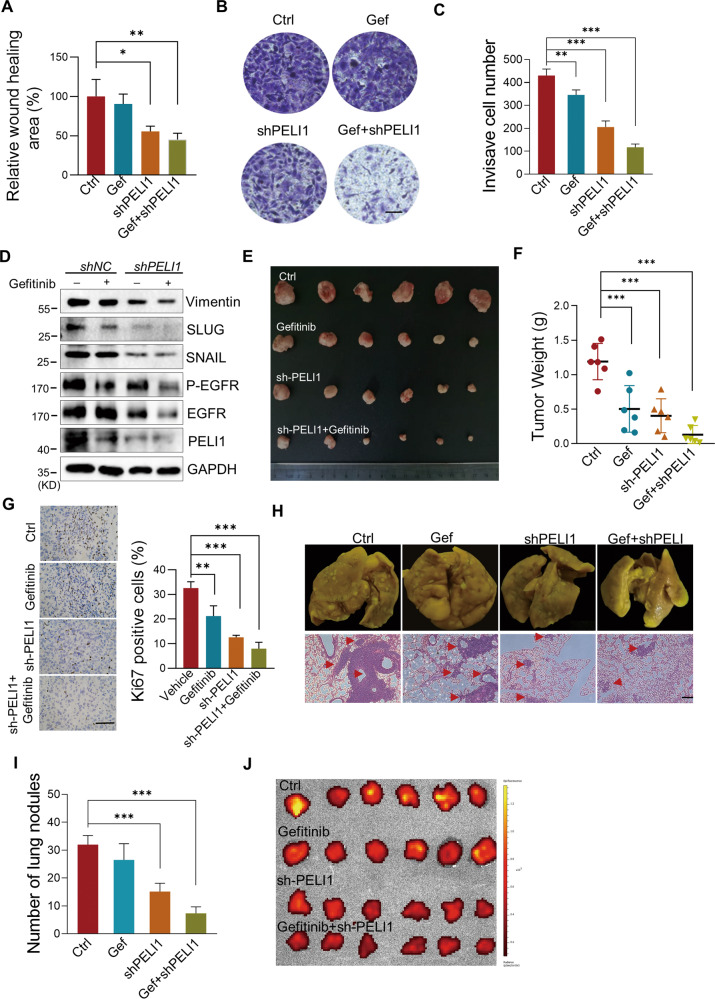
Inhibition of PELI1 and EGFR suppressed breast cancer. **A**–**C** Quantification of the migration (**A**) and invasion (**B**, **C**) of MDA-MB-231 cells transfected with PELI1-shRNA with or without Gefitinib (2 μM) treatment (*N* = 3, scale bar, 100 μm). **D** Western blotting analysis of the indicated proteins with or without PELI1 knockdown and Gefitinib (2 μM) treatment. **E**–**G** Effects of PELI1 knockdown and Gefitinib treatment on the tumor incidence of MDA-MB-231 cells in nude mice. The mice were subcutaneously transplanted with MDA-MB-231/Con-shRNA and MDA-MB-231/PELI1-shRNA cells (5 × 10^6^/mouse) and were treated with or without Gefitinib (50 mg/kg) orally every other day for 2 months. Representative images of tumors (**E**) and tumor weight (**F**) are represented (*N* = 6 per group). The tumors were made into paraffin sections and the Ki67-positive cells (**G**, scale bar, 50 μm) were quantified. **H** Effects of PELI1 knockdown and Gefitinib treatment on the lung-metastasis of MDA-MB-231 cells. NYG mice were injected with MDA-MB-231/Con-shRNA and MDA-MB-231/PELI1-shRNA cells (2 × 10^5^/mouse) via tail vein, and were treated with or without Gefitinib (50 mg/kg) orally every other day for 1 month. The whole indicated lung tissues were stained with Bouin fluid and made into HE stained sections (scale bar, 50 μm)**. I** Quantitative analysis of the metastatic lung nodules in H (*N* = 6 per group). **J** The GFP fluorescence intensity of lung tissues from **H** are shown. *P < 0.05, **P < 0.01, ***P < 0.001. All *P* values were determined by unpaired two-tailed Student’s *t* test.

EMT is required for cancer metastasis, and has closely relationship with EGFR inhibitor resistance [39]. Thus, we next characterized the functional roles of the relationship between PELI1 and EGFR in the EMT progress. In keeping with previous studies, either PELI1 knockdown or Gefitinib treatment inhibited the ability of the migration, invasiveness and tumor spheres formation of MDA-MB-231 cells, which were further strongly enhanced by the combined treatments (Figs. 5A–C and S5A-C). Consistently, knockdown of PELI1 led to additional reduce of EMT-related proteins compared to the treatment of Gefitinib, including Vimentin, SNAIL and SLUG (Fig. 5D). The co-inhibition of PELI1/EGFR also led to a dramatic decrease of Vimentin and increase of E-cadherin, as indicated by immunofluorescence staining (Fig. S5D).

Next, we used the mouse subcutaneous xenograft model to evaluate the effect of combination of PELI1 knockdown and EGFR inhibition on tumor growth. As expected, single inhibition of PELI1 and EGFR led to smaller tumors than controls. Notably, their combination led to further marked reduction in tumors growth (Fig. 5E, F). Furthermore, the immunohistochemical staining of the tumors demonstrated that Ki67 expression, a well-known proliferative marker, was dramatically down-regulated in the tumors from the mice with the combined treatments of PELI1 knockdown and Gefitinib (Fig. 5G).

The efficacy of this combination against breast cancers was further confirmed by major reductions in metastatic capacity of cancer cells in vivo. we found that combination of PELI1 knockdown and EGFR inhibition significantly decreased metastatic potential of cancer cells to lung in caudal vein xenograft models compared to the either single treatment, as demonstrated by reduced number of micrometastatic nodules (Fig. 5H). Similarly, considerable reduce of MDA-MB-231 cells-derived tumor nodules in lung were also detected in mice with both PELI1 knockdown and EGFR inhibition (Fig. 5I), as further confirmed by the decreased GFP fluorescence intensity indicating the MDA-MB-231 cells number in lung (Fig. 5J). Together, these results demonstrated that PELI1 knockdown synergized with EGFR inhibitor and that combined therapy showed superior activity against breast cancer metastasis.

### The compound S62 interrupted the interaction between PELI1 and EGFR to suppress breast cancer metastasis

**Fig. 6:**
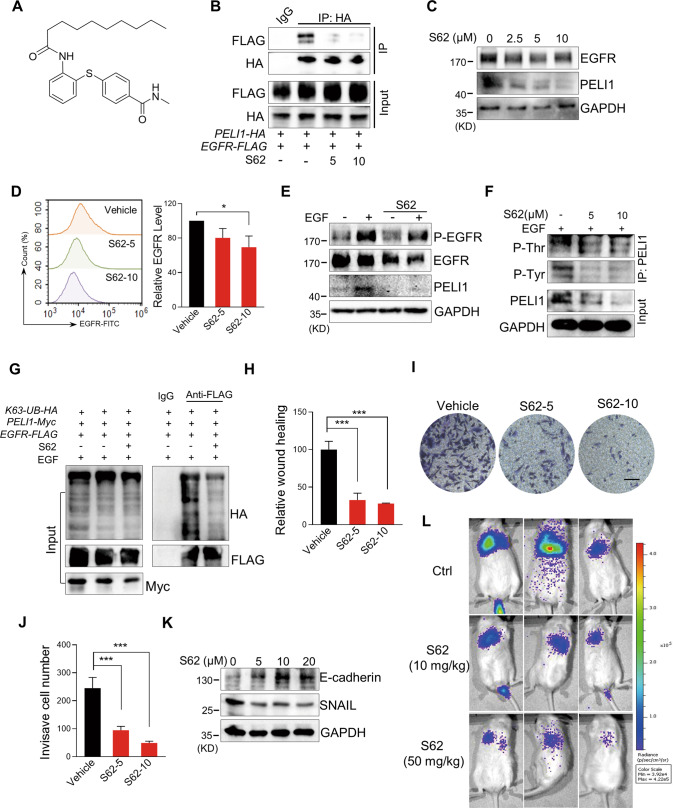
The compound S62 interrupted the interaction between PELI1 and EGFR to suppress breast cancer metastasis. **A** Chemical structure of S62. **B** HEK293T/17 cells with overexpression of *PELI1* and *EGFR* were treated with or without S62 (5 or 10 μM) for 24 h. Co-IP assay was subsequently performed to detect the interaction between PELI1 and EGFR. **C** Western blotting analysis of EGFR and PELI1 in MBA-MB-231 cells with the treatment of S62 for 24 h. **D** Flow cytometric analysis of the membrane EGFR in MDA-MB-231 cells treated with or without S62 for 24 h (*N* = 3). **E** Western blotting analysis of EGFR, PELI1 and phosphorylation of EGFR with or without the treatment of S62 (10 μM) upon EGF stimulation (100 ng/ml). **F** Western blotting analysis of the tyrosine and threonine phosphorylation of PELI1 immunoprecipitated from MDA-MB-231 cells. The cells were treated as in **E**. **G** Western blotting analysis of the K63-mediated polyubiquitination of EGFR immunoprecipitated from HEK293T/17 cells with the treatment of S62 (10 μM). **H** Quantitative analysis of the migration of MDA-MB-231 cells with the treatment of S62 (*N* = 3). **I** Effect of S62 on the invasion of MDA-MB-231 cells. The representative images of invasive cells were shown (scale bar, 100 μm). **J** Quantitative analysis of the invasion of MDA-MB-231 cells in **I** (*N* = 3). **K** Western blotting analysis of E-cadherin and SNAIL in MDA-MB-231 cells with the treatment of S62. **L** The representative images of lung metastasis of breast cancer cells in NYG mice with S62 treatment. MDA-MB-231 transfected with lentivirus that stably expressed luciferase (2 × 10^5^/mouse) were injected into NYG mice via the tail vein. The mice were treated with CMC-Na (0.5%) or S62 (10 or 50 mg/kg) every day for 2 weeks, and then were detected using the bioluminescence imaging. *P < 0.05, ***P < 0.001. All *P* values were determined by unpaired two-tailed Student’s *t* test.

Based on the decrease of PELI1 protein indicated by ELISA assay in MDA-MB-231 cells (Fig. S6A), we screened our in-house small-molecule library of 200 compounds and identified S62 as a potential small molecule disruptor of PELI1/EGFR (Fig. 6A). Compared to the co-immunoprecipitation with each other in control group cells, S62 treatment impaired the interaction of PELI1 and EGFR (Fig. 6B). Indeed, both protein levels of PELI1 and EGFR were greatly reduced after S62 treatment in MDA-MB-231 cells (Figs. 6C and S6B). S62 treatment also led to a decrease of the membrane EGFR (Fig. 6D), whereas it failed to block the phosphorylation of EGFR upon EGF stimulation (Fig. 6E). Furthermore, S62 reduced the tyrosine and threonine phosphorylation of PELI1 and K63-linked ubiquitination of EGFR (Fig. 6F, G).

Next, we evaluated the effects of S62 against breast cancers. Similar with the findings from the combination of PELI1 knockdown and EGFR inhibition, S62 significantly inhibited the migration and invasiveness of MDA-MB-231 cells (Figs. 6H–J and S6C). Moreover, immunoblotting demonstrated that S62 treatment led to a dose-dependent increase of E-cadherin protein level but a decrease of SNAIL (Fig. 6K). We then examined the MDA-MB-231 cells-derived tumor nodules in lung after caudal vein injection, and found that S62 inhibited the metastasis of MDA-MB-231 cells as indicated by the decreased luciferase intensity in lung (Fig. 6L).

Indeed, S62 showed mild effect on the cell viability of tumor cells or normal cells (Fig. S6D–K) and even the MDA-MB-231 cells sensitivity to Gefitinib (Fig. S6L). On the other hand, overexpression of PELI1 did not reverse the suppressed migration of MDA-MB-231 cells caused by S62 treatment (Fig. S6M). These results indicated that S62 functions as a disruptor of PELI1 and EGFR interaction but not binding to PELI1 or EGFR alone.

## Discussion

**Fig. 7:**
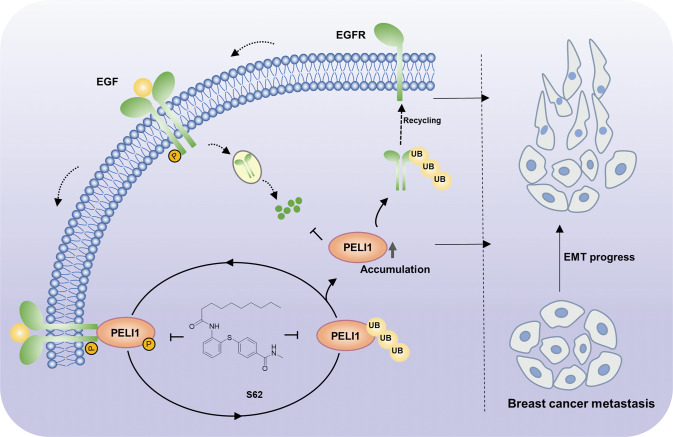
Schematic diagram illustrates that PELI1 and EGFR cooperated to promote breast cancer metastasis. EGFR acts as a phosphokinase of PELI1 to promote its phosphorylation, leading to the E3 ubiquitin ligase activation. Simultaneously, PELI1 enhanced the stability of EGFR via the K63-linked polyubiquitination. Compound S62 as a small molecule disruptor of PELI1/EGFR repressed breast cancer metastasis.

EMT is one of the critical mechanisms in breast cancer metastasis [5, 40]. Abnormal regulation of EGFR signaling in tumors usually leads to changes in cell proliferation and adhesion, increasing cell aggressiveness and motility, which are hallmarks of EMT and the initial stages of tumorigenesis [41]. It has been reported that EGFR is involved in regulating EMT in prostate cancer cells [42], pancreatic cancer cells [43], and colorectal cancer cells [44]. However, the single-targeted anti-EGFR therapies have limited efficacy in the clinical and preclinical treatment of TNBC [45, 46]. In our study, we identified a novel regulation mechanism by which PELI1 and EGFR cooperate to promote breast cancer metastasis (Fig. 7). PELI1 is positively correlated with EGFR expression in breast cancers. The co-inhibition of the PELI1 and EGFR effectively suppressed the migration, invasion and tumor sphere formation ability and metastasis of MDA-MB-231 cells.

The molecular mechanism underlying the high expression and oncogenic roles of PELI1 in cancers remains limited. It had been documented that PELI1 plays a protective role in tumor development mainly by regulating K63-linked polyubiquitination of the substrate proteins, including SNAIL/SLUG. Our study revealed that PELI1 interacts with and stabilizes EGFR via K63-linked ubiquitination, leading to the enhanced activation of EGFR due to the accumulation of intracellular and membrane EGFR (Fig. 7). EGFR recycling plays a crucial role in tumor development [36]. It has been reported that GOLM1 selectively interacts with EGFR and assists EGFR recycling back to the plasma membrane to drive hepatocellular carcinoma metastasis [35]. EGFR recycling is also regulated by ISGylation leading to the more aggressive tumor behaviors observed in breast cancer [47]. Besides these regulations, several ubiquitin ligases were also reported to be involved in EGFR trafficking and recycling such as CHIP and SMURF2 [48, 49]. In line with these previous findings, we revealed that PELI1 acts as an E3 ubiquitin ligase of EGFR to enhance the recycling of EGFR, which accounts for its aberrant expression in triple-negative breast cancers.

We also explored the mediatory roles of EGFR in the PELI1 expression. Multiple phosphorylation sites of PELI1 have been previously identified, several of which were required for its activation of E3 ubiquitin ligase [50]. However, the mechanism of PELI1 activation remains poorly understood. Indeed, Ser-76, Ser82, and Thr-86 have been proven to be uniquely phosphorylated by interleukin-1 receptor-associated kinase 1 (IRAK1) and IRAK4, while Ser-78, Thr-80, Thr-288 and Ser-293 are phosphorylated by other IRAKs and TANK-binding kinase 1 [38, 51]. These findings imply that the phosphorylation sites in PELI1 that is critical for activation are dependent on the upstream phosphokinases. Our study demonstrated that EGFR activation led to the upregulated phosphorylation and subsequent activation of PELI1. Given that EGFR is a tyrosine kinase receptor, EGFR appears to be a phosphokinase of PELI1 that phosphorylates PELI1 at Tyr154, leading to its phosphorylation at Thr264 and auto-ubiquitination at Lys169. However, we cannot exclude the possibility that EGFR recruits an intermediate protein to phosphorylate and activate PELI1 leading to the autoubiquitylation. Therefore, PELI1-EGFR interactions lead to bidirectional communication and to the formation of a regulatory loop system. EGFR phosphorylates and activates PELI1. Meanwhile, EGFR undergoes PELI1-mediated ubiquitylation and protection from degradation, which results in the enhancement of downstream signaling of EGFR.

For most patients with mutant *EGFR*, the initial efficacy of EGFR-TKI is verified, but drug resistance inevitably occurs with the extension of drug application time [52]. Although targeting-EGFR immunotherapy is encouraging and promising, it is usually not effective due to the molecular characteristics and acquired drug resistance mechanism [53, 54]. In addition, EMT is associated with EGFR-TKI resistance in EGFR-mutated cancers [55, 56]. Our data showed that knockdown of PELI1 enhanced the sensitivity of EGFR inhibitor against the EMT in breast cancer cells through a decrease of EGFR levels, suggesting that co-inhibition of PELI1 and EGFR have a synergetic effect with EGFR-TKI. Accordingly, we identified a compound S62 as a potential small molecule disruptor of PELI1/EGFR. Our data indicated that S62 interfered the PELI1-EGFR interactions leading to the reduced phosphorylation of PELI1 and membrane EGFR. We also confirmed that S62 effectively repressed breast cancer metastasis as indicated by the reduced migration and invasion of MDA-MB-231 cells in vitro and in vivo, providing a potent leading compound for the development of novel targeting therapeutics for TNBC metastasis.

Taken together, we demonstrated that PELI1 and EGFR cooperated to promote breast cancer metastasis. Our study provides mechanistic insights and therapeutic interventions for breast cancer metastasis. Based on the commonly high-expression of PELI1 in cancers, the co-inhibition of the PELI1-EGFR may also repress the metastasis in other cancers.

## Materials and methods

### Cell culture

MDA-MB-231, NCI-H1975, NCI-H1650, HCT-116 and HEK293T/17 cell lines were obtained from National Collection of Authenticated Cell Cultures. HepG2 and HL-7702 cell lines were stored by our laboratory. MDA-MB-231 cells were cultured in L-15 medium (Gibco, CA, USA) with 10% FBS at 37 ℃. NCI-H1975, NCI-1650 and HL-7702 cells were cultured in RPMI-1640 medium (BI, Beit-Haemek, Israel). The HCT-116 cells were cultured in McCoy’s 5A medium (BI, Beit-Haemek, Israel). HepG2 and HEK293T/17 were cultured in DMEM medium (BI, Beit-Haemek, Israel). These cells were maintained with 10% fetal bovine serum at 37 ^o^C and 5% CO_2_.

### Western blot and antibodies

Western blotting was performed as described previously [12]. All the antibodies used in this study were provided in Table S2.

### Plasmid construction and lentivirus transfection

The PELI1 truncation plasmids were inserted into pCDNA3.1-HA vector, including FL (full length, amino acids 1–418), P1 (amino acids 1–287), P2 (amino acids 1–200), P3 (amino acids 80–287), P4 (amino acids 288–418). The EGFR truncation plasmids were inserted into pLVX-FLAG-Puro vector, including FL (full length, amino acids 1–1210), E1 (amino acids 1–668), E2 (amino acids 669–979), E3 (amino acids 712–979), E4 (amino acids 980–1210). The point mutation plasmids *PELI1*-Y154F, *PELI1*-T175A, *PELI1*-T264A, and ubiquitin plasmids K48-*UB*, K63-*UB* were inserted into pCDNA3.1-HA vector. The *UB*-WT were inserted into pRK5-HA vector. The PELI1 overexpression plasmid and the point mutation plasmid *PELI1*-K264R were inserted into pCDNA3.1-Myc vector. These plasmids were purchased from Miaolingbio company. The lentivirus overexpression plasmids PELI1 and Luciferase were constructed in pCDH-CMV-HA-Puro vector, and the lentivirus interference plasmids sh-PELI1-1, sh-PELI1-2 were constructed in pLKO.1-Puro vector (Table S3). The *EGFR* siRNA-1 (stB0004798A) and *EGFR* siRNA-2 (stB0004798B) were purchased from Guangzhou RIBOBIO company.

To prepare the lentivirus, HEK293T/17 cells were transfected with plasmids by using LipoFiter^TM^ transfection reagent (Hanbio Biotechnology, Shanghai, China). In all, 50% PEG8000 and 5 M sodium chloride were added into the supernatant after filtration of cells. The lentivirus precipitation was collected and resuspended with PBS after incubation at 4 °C overnight. Then the cells were infected with the lentivirus and 8 μg/ml protamine for the protein overexpression or knockdown.

### Immunohistochemistry and immunoprecipitation assays

At the first, the tissue microarray was dewaxed and hydrated at room temperature. Then it was heated in antigenic repair solution for 15 min and incubated in 3% hydrogen peroxide. Then the tissue microarray was incubated with primary antibody after being covered goat serum and put it in a wet box at 4 °C overnight. On the second day, the tissue microarray was incubated with the second antibody and streptavidin–peroxidase solution. After that, the tissue microarray was dyed by DAB solution and hematoxylin. Finally, the tissue microarray was placed in acid ethanol differentiation solution (1%) for 3 s and sealed with neutral balsam. The tissue microarray was scanned by a panoramic slide scanner and quantitatively analyzed by Quant Center software. *H*-Score values were calculated by the following:

H-Score = (percentage of weak intensity × 1) + (percentage of moderate intensity × 2) + (percentage of strong intensity × 3).

After successful transient transfection of HEK293T/17 cells, the cells were lysed in 550 μl Co-IP lysis buffer. The 50 μl of supernatant was input after centrifugation and the other was incubated with antibody conjugated to agarose beads at 4 °C overnight. The complexes were washed and denaturized at 98 °C for 10 min for western blotting analysis.

### Immunofluorescence analysis

The cell suspension was seeded into the 12-well culture plate with round cover slices. Immunofluorescence experiment was performed after 24 h. The Cell membranes were fragmented with 0.1% Triton X-100 in PBS after fixation with 4% formaldehyde and were blocked with 3% BSA. The slices were incubated with the primary antibody and fluorescent secondary antibodies, and then were sealed with anti-fluorescence quencher (with DAPI). To the end, the cell image was immediately observed under confocal microscope or stored at 4 °C.

### RNA isolation and quantitative real-time PCR

RNA isolation and quantitative real-time PCR were performed as previously described [57]. The related primers were provided in Table S3.

### Cell migration and invasion assays

Cell migration was tested by wound healing assay. After drug treatment, cell supernatants were discarded and PBS washed cells three times. Then cells were scratched by a peptide and incubated in medium with 2% fetal bovine serum (FBS). The wound healing area was observed and calculated. A transwell assay was carried out to analyze cell invasion ability. The lower surface of the 0.8 μm-transwell chambers were covered with 30 μl fibronectin (10 μg/ml, Corning, NY, USA) and air-dried at room temperature. The Matrigel (Corning, NY, USA) diluted 1:30 in medium were added in the surface of transwell chamber. Then cell suspension with serum-free culture medium were inoculated into the transwell chamber. The cells were stained with crystal violet hydrate solution (Sigma, NY, USA) after fixation and counted under inverted microscope.

### Tumor sphere assay

The 5 μl thrombin (0.1 U/μl) (Sea Run Holdings, Maine, USA) were added into the 24-well plates. Then a blend of the cell suspension and an equal volume of the fibrinogen (Solarbio, Beijing, China) was seeded into the 24-well plate (1000 cells/well) and mixed with thrombin. The plates were placed in incubator for 10 min until the mixture into semi-solid and 1 ml culture medium was replenish. After incubation for 7 days, the tumor spheres were formed and analyzed.

### Animal studies

The antitumor assay was performed by xenograft mouse model. Breast cancer cell suspension (5 × 10^6^ cells/mouse) mixed with the same volume of Matrigel were injected subcutaneously into BALB/C-nu/nu mice (Vital River Laboratories, Beijing, China). The mice were treated with vehicle 0.5% CMC-Na or Gefitinib (50 mg/kg) every other day for eight weeks. To detect the effect of compound S62 on tumor metastasis, MDA-MB-231 cells stably expressed luciferase were injected into NYG mice (Changsheng biotechnology, Liaoning, China) via tail. The mice were treated with vehicle 0.5% CMC-Na or S62 compound and monitored by in vivo bioluminescence imaging system after 2 weeks. PELI1 tissue-specific knockout mice were generated by mating PELI1 flox/flox mice (NBRI, Nanjing, China) with MMTV-Cre mice (Shanghai Model Organisms Center, Shanghai, China). The female 4–6 weeks mice were randomly assigned to experimental groups.

Mice were maintained in sterilized animal facility with sterilized food, and water. All animal studies were performed in accordance with the Guidelines for the Care and Use of Laboratory Animals and were approved by the Institutional Animal Care and Use Committees at Shandong University.

### Statistical analysis

The data were analyzed by the Prism 8 software (GraphPad) and presented as mean ± standard deviation (SD). The comparison was performed by Student’s *t* test or one-way analysis of variance. *P* value <0.05 was considered statistically significant.

## Supplementary information

Supplementary information

Table S1

## Data Availability

All data in this study will be available from the corresponding author on reasonable request.
