# Supplementary material for: PELI1 and EGFR cooperate to promote breast cancer metastasis
Source: Oncogenesis. 2023 Feb 25;12(1):9. doi: 10.1038/s41389-023-00457-3 (PMC9968314; doi:10.1038/s41389-023-00457-3)
Supplement: Supplementary file 1 — Supplementary information [file 41389_2023_457_MOESM1_ESM.pdf]

Supplementary Information for

Original article

PELI1 and EGFR cooperate to promote breast cancer metastasis

Jie Qi[1,2], Guangsen Xu[1,2], Xiaoxia Wu[1,2], Chunhua Lu[1,2], Yuemao Shen[1,2*], Baobing Zhao[1,2,3*]

[1]Key Lab of Chemical Biology (MOE), School of Pharmaceutical Sciences, Cheeloo College of Medicine, Shandong University, Jinan, Shandong, 250012, China

[2]NMPA Key Laboratory for Technology Research and Evaluation of Drug Products, School of Pharmaceutical Sciences, Cheeloo College of Medicine, Shandong University, Jinan, Shandong 250012, China

[3]Department of Pharmacology, School of Pharmaceutical Sciences, Cheeloo College of Medicine, Shandong University, Jinan, Shandong, 250012, China

* Correspondence

Yuemao Shen (yshen@sdu.edu.cn)

Baobing Zhao (baobingzh@sdu.edu.cn)

Supplementary Figures

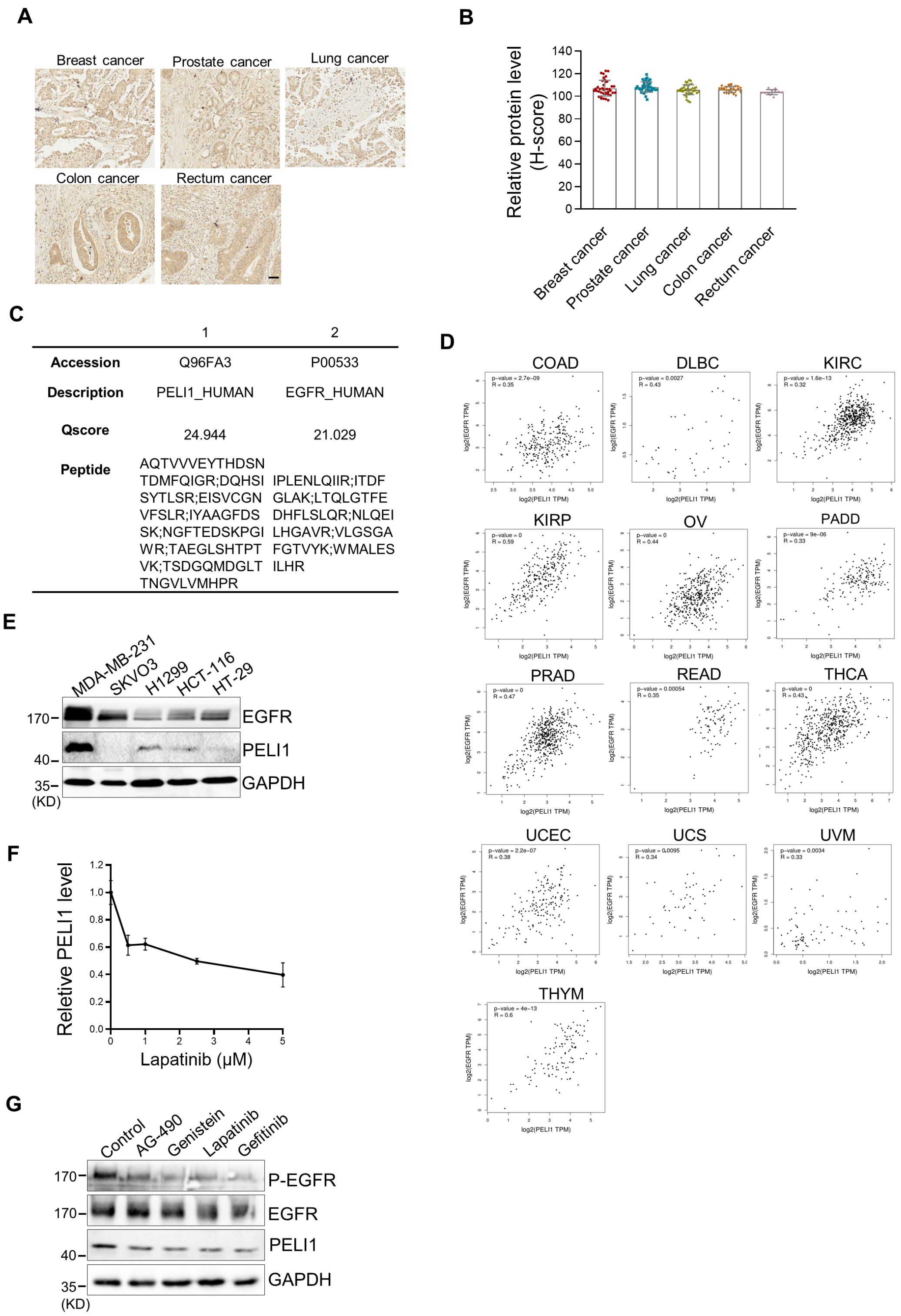

Figure S1. PELI1 has a close correlation with EGFR. (A-B) IHC analysis of PELI1 expression in tissue microarray with five kinds of cancers patients, including breast cancer, prostate cancer, lung cancer, colon cancer and rectum cancer. The representative

images of IHC staining (A, scale bar, 50 μm) and quantitative analysis of PELI1 level. **(C)** EGFR was identified from the co-immunoprecipitation with PELI1 by MS analysis. **(D)** Correlation analysis of *PELI1* and *EGFR* genes in different cancer from the GEPIA online database. The correlation rates and *P* value are shown. **(E)** Western blotting analysis of the levels of PELI1 and EGFR proteins in five kinds of cancer cell lines. GAPDH was used as a loading control. **(F)** ELISA analysis of the change of PELI1 in MDA-MB-231 cells with Lapatinib. **(G)** Western blotting analysis of the abundance of PELI1 with the treatment of four EGFR inhibitors (5 μM). GAPDH was used as a loading control.

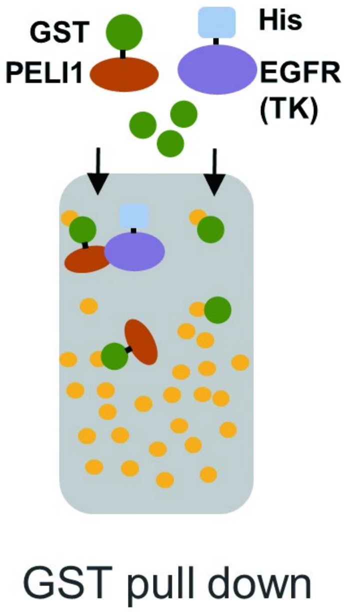

Figure S2. The sketch map of GST-pull down assay. Complete PELI1 protein (with GST tag) and partial intracellular EGFR protein (with His tag) were purified and the GST agarose beads bound to PELI1 proteins or GST protein. Then PELI1 protein or GST protein incubated with intracellular EGFR protein and the complex were subjected to western blotting after washing.

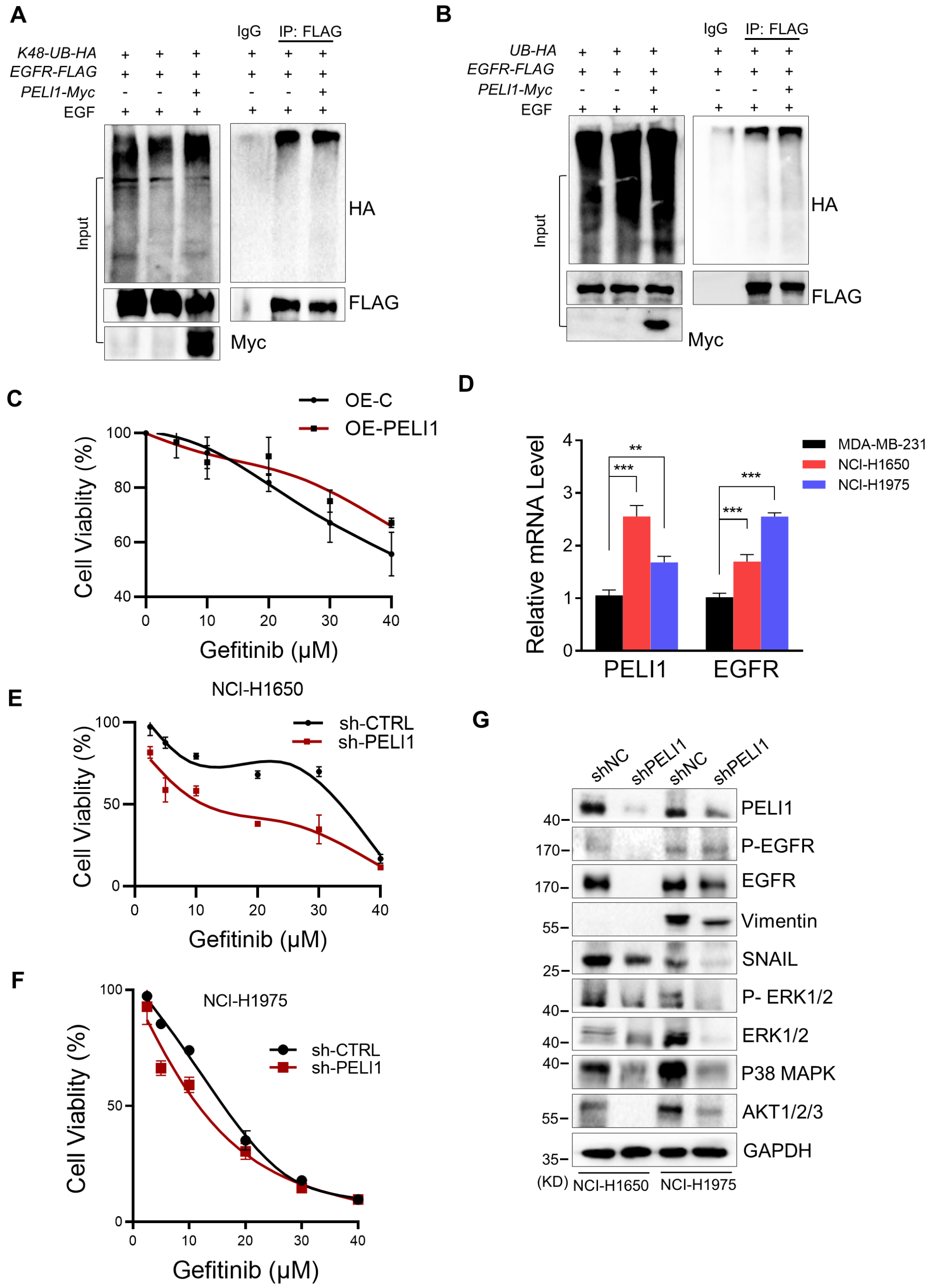

Figure S3. Inhibition of PELI1 increases the sensitivity of EGFR-mutated cells to Gefitinib. (A) Immunoblotting analysis of the change of K48-linked polyubiquitination of EGFR with PELI1 overexpression in HEK293T/17 cells upon EGF (100 ng/ml) stimulation. **(B)** Immunoblotting analysis of the change of total ubiquitination of EGFR with PELI1 overexpression in HEK293T/17 cells upon EGF (100 ng/ml) stimulation. **(C)** Effect of PELI1 overexpression on Gefitinib sensitivity was detected in MDA-MB-

231 cells. **(D)** The mRNA level of *PELI1* was evaluated by qRT-PCR in MDA-MB-231, NCI-H1650 and NCI-H1975 cells. **$P < 0.01$, ***$P < 0.001$. **(E-F)** Effect of PELI1 knockdown on Gefitinib sensitivity was detected in NCI-H1650 (E) and NCI-H1975 cells (F). **(G)** Western blotting analysis of the levels of EMT related proteins and EGFR signaling downstream proteins in NCI-H1650 and NCI-H1975 cells with PELI1 knockdown.

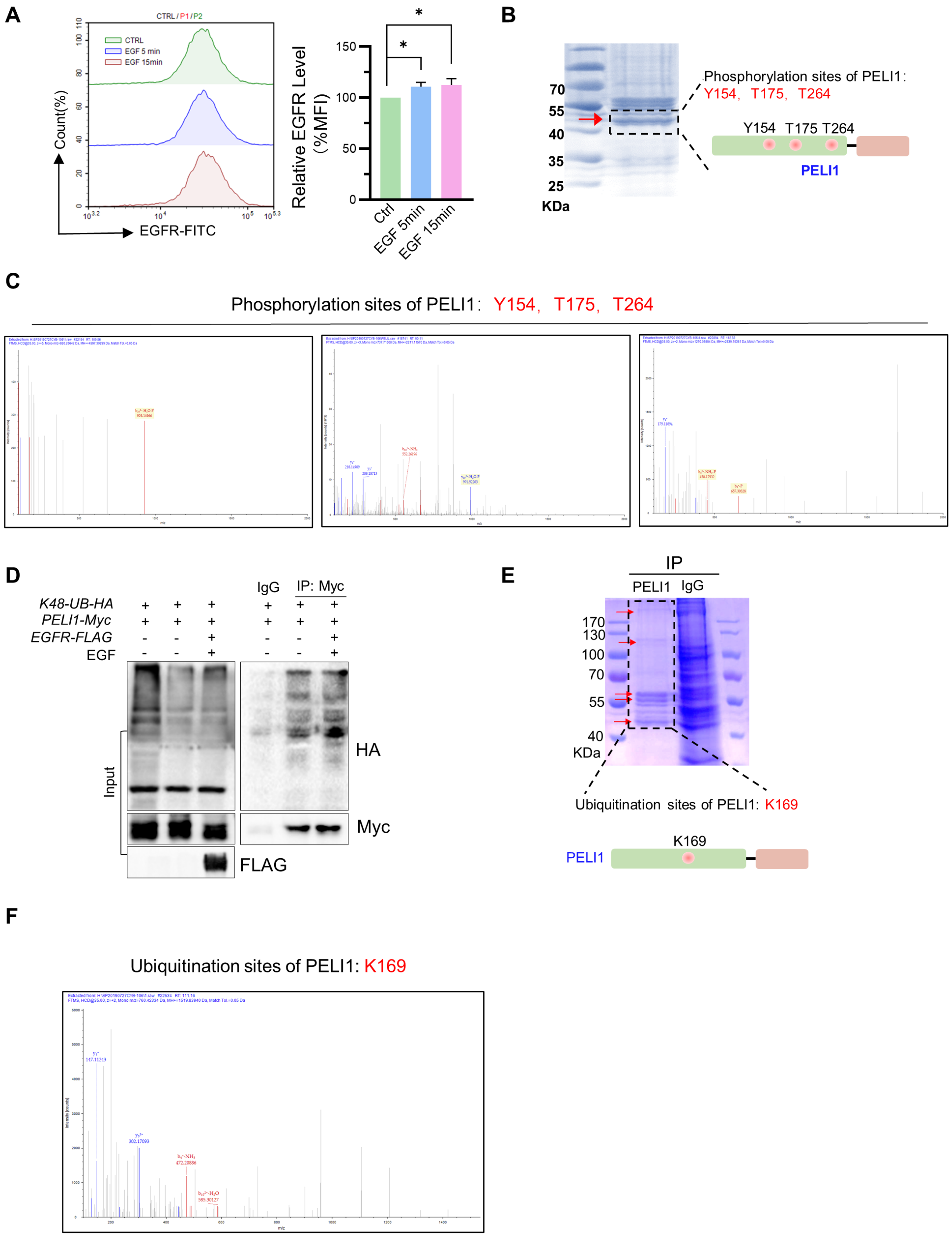

Figure S4. The mass spectrometry analysis of phosphorylation sites and ubiquitination site of PELI1 protein. (A) Flow cytometric assay of the membrane EGFR in MDA-MB-231 cells treated with EGF (100 ng/ml) for 5, 15min. **P* < 0.05. **(B-C)** MS analysis of phosphorylation sites of PELI1. Whole cell lysates from MDA-MB-231cells were immunopurified with anti-PELI1 antibody and analyzed by MS. The

proteins were measured by Coomassie Brilliant Blue staining (B), and the spectrum represented the phosphorylation sites of PELI1 which were identified by MS (C). **(D)** Immunoblotting showed the K48-linked polyubiquitination of PELI1 with EGFR overexpression in HEK293T/17 cells. **(E-F)** MS analysis of ubiquitination site of PELI1. MDA-MB-231 cells were treated as in B and the extracts were measured by Coomassie Brilliant Blue staining (E), and the spectrum represented the ubiquitination site of PELI1 (F).

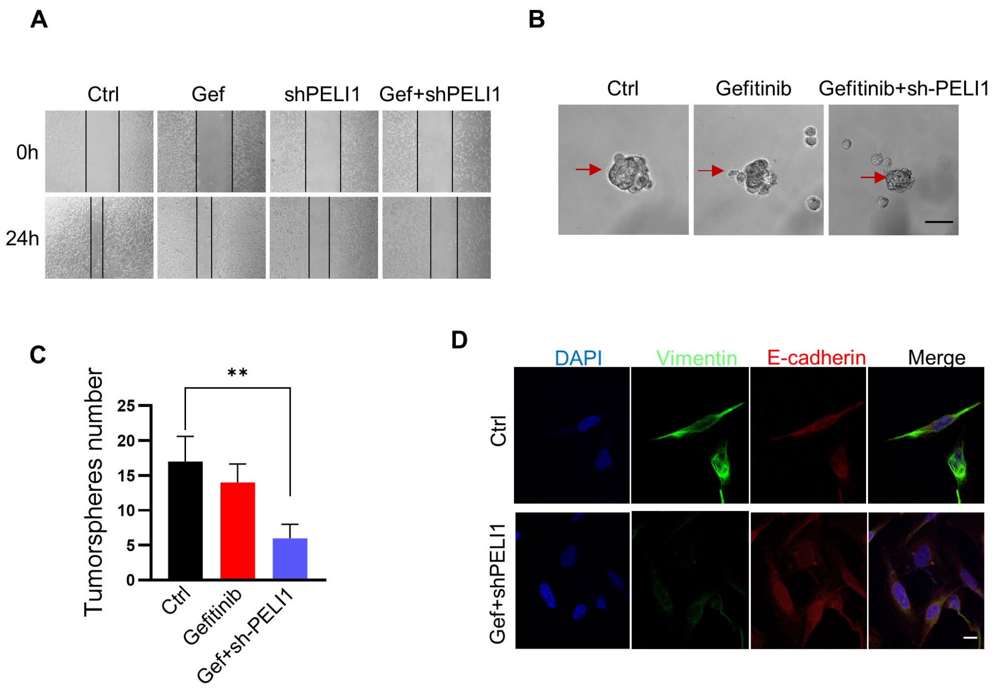

Figure S5. Inhibition of PELI1 and EGFR further reduces the tumor spheres formation and EMT process. (A) The representative images of MDA-MB-231 migration cells with or without PELI1 knockdown and Gefitinib (2 μM) treatment. **(B-C)** The formation of tumor spheres (scale bar, 50 μm) in MDA-MB-231/con-shRNA and MDA-MB-231/PELI1-shRNA cells were detected with Gefitinib (2 μM) treatment. $^{**}P < 0.01$. **(D)** The confocal microscopy showed the levels of Vimentin and E-cadherin with PELI1 knockdown and Gefitinib (2 μM) treatment in MDA-MB-231 cells (scale bar, 10 μm).

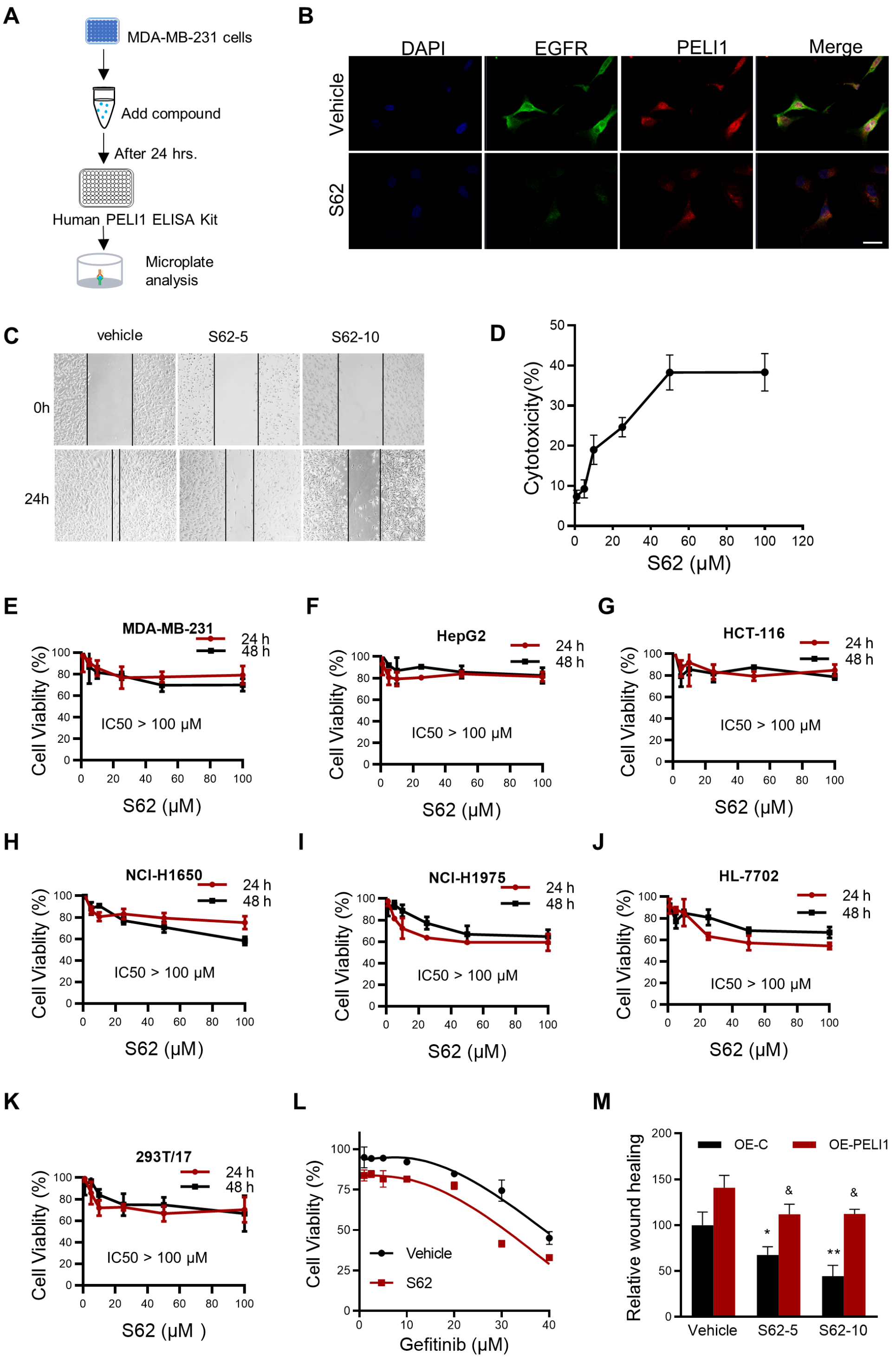

Figure S6. The S62 compound which is screened by ELISA has less cytotoxicity. **(A)** The sketch map of screening inhibitors of PELI1 by ELISA assay. **(B)** IF analysis showed the levels of PELI1 and EGFR with S62 (10 μM) treatment in MDA-MB-231 cells (scale bar, 10 μm). **(C)** The representative images of the migration of MDA-MB-231 cells with the treatment of S62. **(D)** The cytotoxicity of S62 was detected by LDH

release assay. **(E-K)** The cell viability of indicated cells with S62 treatment was tested by CCK-8 assay. **(L)** Effect of S62 (10 μM) treatment on Gefitinib sensitivity was tested in MDA-MB-231 cells. **(M)** The quantification of migration ability was detected in MDA-MB-231 cells with PELI1 overexpression and S62 (5 or 10 μM) treatment for 24 h. $^{*}P < 0.05$, $^{**}P < 0.01$, $^{\&}P < 0.05$.

Supplemental Tables

Table S1. GO and KEGG enrichment analysis of proteins from co-immunoprecipitation with PELI1 in MDA-MB-231 cells (*P* value <0.05).

Table S2. Antibodies and commercial reagents for this study.

Antibodies/Reagents	Source	Catalog number
Vimentin	CST	5741
E-cadherin	CST	3195
SNAIL	CST	3879
SLUG	CST	9585
HA-tag	CST	3724
Phospho-EGFR	CST	3777T
Rabbit IgG	CST	7074
Mouse IgG	CST	7076
ERK1/2	CST	4695
phospho-ERK1/2	CST	4370
P38 MAPK	CST	9218
Pellino-1	CST	31474S
Myc-tag	CST	2276S
EGFR (Alexa Fluor® 488 Conjugate)	CST	5616
β-Tubulin	Abmart	M30109S
GAPDH	Abcam	ab128915
Pellino-1	Abcam	ab199336
EGFR	Abcam	Ab30
Flag-tag	Abcam	Ab205606
AKT	Abcam	ab200193
Phosphotyrosin	Abcam	ab179530
Phosphothrenine	Abcam	ab9337
Myc-tag	MBL	562
Flag-tag	MBL	M185-3
HA-tag	MBL	M180-3
Protein A/G-Agarose	Santa	sc-2003
Gefitinib	MCE	HY-50895
Lapatinib	MCE	HY-50898
AG-490	MCE	HY-12000
Genistein	MCE	HY-14596
PrimerScript RT reagent	Takara	RR047A
SYBR Premix Ex Taq	Takara	RR420A
PELI1 ELISA Kit	EK-Bioscience	EK-H12455
GST-tag protein purification kit	Beyotime	P2262
Chip with five tumor tissues	Alenabio	XMC-2081b
Chip with breast tumor tissues	OUTDO BIOTECH	HBreD140Su07

Table S3. Primer sequences list for this study.

Gene	Primer sequences
PELI1 (Human)	Forward: CAGCACTGTGCATATTGCTTG
	Reverse: CGGCCAATCTGAAACATATCGG
EGFR (Human)	Forward: AGGCACGAGTAACAAGCTCAC
	Reverse: ATGAGGACATAACCAGCCACC
GAPDH (Human)	Forward: GGAGCGAGATCCCTCCAAAAT
	Reverse: GGCTGTTGTCATACTTCTCATGG
sh-PELI1-1	CCAGAACTGTTTGGTTGATTA
sh-PELI1-2	GCAAAGGTCAACACAGTATAT
